# PHYSICAL ACTIVITY AND SEDENTARY BEHAVIOR IN RESIDENTS OF ASSISTED LIVING: A PILOT LONGITUDINAL STUDY

**DOI:** 10.1093/geroni/igae098.2999

**Published:** 2024-12-31

**Authors:** Jung Yoen Son, She’Lon Tucker, Carol Vos, Janet Larson

**Affiliations:** University of Michigan, Ann Arbor, Michigan, United States; University of Michigan, Ann Arbor, Michigan, United States; University of Michigan, Ann Arbor, Michigan, United States; University of Michigan, Ann Arbor, Michigan, United States

## Abstract

Low levels of physical activity (PA) and high sedentary behavior (SB) place older assisted living (AL) residents at risk for functional decline and premature transfer to a higher level of care. This study aimed to examine the feasibility and acceptability of conducting a longitudinal study using two objective measures of PA and SB. We recruited participants from four AL facilities in Michigan. Participants wore two devices (ActiGraph at the wrist and ActivPal on the anterior thigh) for seven consecutive days twice, once at baseline and again six months later, and completed questionnaires. We evaluated recruitment, retention, and compliance with and acceptability of wearing devices, and conducted a longitudinal multilevel model to estimate preliminary changes in PA and SB over 6 months. Forty-two people volunteered, 19% were excluded with a low Mini-Cog score, 62% completed baseline (77% female; mean age=80.1), and 60% completed 6 months data collection. Most participants were willing to perform two measures of PA and SB and generally found the procedures to be acceptable. Over 6 months, moderate to vigorous PA significantly declined by 8 min/day (p=0.02), light PA declined by 18 min/day (p=0.13), and SB increased by 15 min/day (p=0.48). In conclusion, the results support the feasibility and acceptability of this research protocol. Participants had a small decline in PA over a six-month period of time, which was not clinically meaningful but could become significant if the trend continued.

